# Pullulan-Graft-Polyoxazoline: Approaches from Chemistry and Physics

**DOI:** 10.3390/molecules29010026

**Published:** 2023-12-19

**Authors:** Ivan M. Zorin, Petr A. Fetin, Nina G. Mikusheva, Alexey A. Lezov, Igor Perevyazko, Alexander S. Gubarev, Anna N. Podsevalnikova, Sergey G. Polushin, Nikolai V. Tsvetkov

**Affiliations:** 1 Institute of Chemistry, Saint-Petersburg State University, Universitetskaya Nab. 7/9, 199034 Saint-Petersburg, Russia; p.fetin@spbu.ru; 2 Department of Molecular Biophysics and Polymer Physics, Saint-Petersburg State University, Universitetskaya Nab. 7/9, 199034 Saint-Petersburg, Russia; n.mikusheva@spbu.ru (N.G.M.); a.a.lezov@spbu.ru (A.A.L.); i.perevyazko@spbu.ru (I.P.); a.gubarev@spbu.ru (A.S.G.); a.podsevalnikova@spbu.ru (A.N.P.); s.polushin@spbu.ru (S.G.P.)

**Keywords:** pullulan, POx, polyoxazoline, graft copolymer, click chemistry

## Abstract

An approach to the preparation of pullulan-graft-poly(2-methyl-2-oxazoline)s based on Cu-catalyzed azide–alkyne cycloaddition with polyoxazoline-azide was applied. All of the obtained polymers were characterized through classical molecular hydrodynamic methods and NMR. The formation of graft copolymers was accomplished by oxidative degradation of pullulan chains. Nevertheless, graft copolymers were obtained as uniform products with varied side chain lengths and degrees of substitution.

## 1. Introduction

Pullulan is a water-soluble, neutral, linear, glucosic polysaccharide commonly produced by strains of the polymorphic fungus *Aureobasidium pullulans*, first reported in 1938 [1] and first isolated in 1958 [2].

Since that time, pullulan has found versatile applications in the food industry [3,4,5], pharmaceutics [6,7,8], and even in environmental remediation agents [9,10]. Due to its non-toxicity, biodegradability, antioxidant qualities, film-forming ability, blood compatibility, mucosal adhesion, etc., pullulan has been found to be a promising agent in biomedical applications [11,12,13,14,15]. However, these are partly limited by its insolubility in organic solvents, mechanical characteristics, and lack of macromolecule-carrying-capable groups [16,17,18].

Chemical modifications of pullulan (or other polysaccharides) allow the benefits of natural polymers to be combined with improved physicochemical properties [19] and can even be tailored for specific forms of delivery [20,21,22]. In biomedicine, pullulan derivatives have been reported to find uses in drug and gene targeting [23].

Another well-known biocompatible class of polymers, namely poly(2-oxazoline)s, has tremendous potential for use in medicine [24,25]. The most common hydrophilic poly(2-oxazoline)s—poly(2-methyl-2-oxazoline) (PMeOx) and poly(2-ethyl-2-oxazoline) (PEtOx)—have demonstrated outstanding properties, including high stability in the physiological pH range, chemical and physical versatility, stealth behavior, antifouling characteristics, and good renal clearance [26,27,28,29]. PEtOx-based polymer–drug conjugates and hemostatic materials have reached human clinical trials [24,30,31,32]. At the same time, PMeOx has been shown to be more hydrophilic than both PEtOx and poly(ethylene glycol) (PEG), exhibits better antifouling properties [33], and allows for higher hydrophobic drug loading [34]. A recent study on PMeOx using X-ray scattering established the interesting fact that poly(2-methyl-2-oxazoline) is not completely amorphous [35]. Conformation of PMeOx under physiological conditions has been reported recently [36]. Poly(2-oxazoline)s are considered to be appropriate alternatives to PEG, since PEG antibodies are being reported repeatedly [37,38,39].

Block copolymers, including blocks of polyoxazoline and low-molecular-weight pullulan, are described in [40,41]. Despite the hydrophilicity of both blocks, such polymers have demonstrated the ability to form micellar aggregates in diluted aqueous solutions, which can be used in the creation of nanoreactors or for drug delivery.

The coating of nanoparticles with various hydrophilic chains, e.g., poly(N-isopropyl acrylamide), poly(vinylamine), poly(methacrylic acid), or low-molecular-weight polyethylene glycol, is a known way to give them mucoadhesive properties [42,43]. The functionalization of silica nanoparticles with PEtOx has also been reported [44]. At the same time, nanoparticles functionalized with PMeOx were shown to penetrate through mucosal tissue considerably more effectively than particles functionalized with PEtOx [45].

A recent study has demonstrated scientific interest in the synthesis of polysaccharide–poly(2-oxazoline) conjugates for improved drug delivery in ophthalmology [46]. In [47], PMeOx was utilized to functionalize hyaluronic acid. The grafting of short chains of PEtOx and other non-ionic polymers to a chitosan backbone, with subsequent formation of mucus-penetrating nanoparticles, has been reported in [48]. We would like to emphasize that, in the last three aforementioned papers, the authors reported the synthesis of graft copolymers containing low-molecular-weight poly(2-oxazoline) chains connected to a single polysaccharide molecule. Such an architecture of the polysaccharide–polyoxazoline conjugate can be useful in the design of mucoadhesive polymers for local drug delivery [48,49].

Thus, all things considered, the present study is dedicated to the modification of pullulan through poly(2-oxazoline) side chains, primarily poly(2-methyl-2-oxazoline), to obtain a novel polysaccharide-based graft copolymer via a “grafting-to” strategy. The fact that both pullulan and PMeOx are well-studied polymers allows us to draw unambiguous conclusions regarding the results of grafting in terms of molecular architecture and molecular hydrodynamics characteristics. In addition to obtaining modified pullulan, successful modification of pullulan through poly(2-oxazoline) side chains will make it possible to use the same method for the well-defined modification of other polysaccharides by poly(2-oxazoline)s, even if the initial polysaccharide has a wider molecular weight distribution, or obtaining its molecular characteristics is difficult for other reasons.

**Figure 1:**
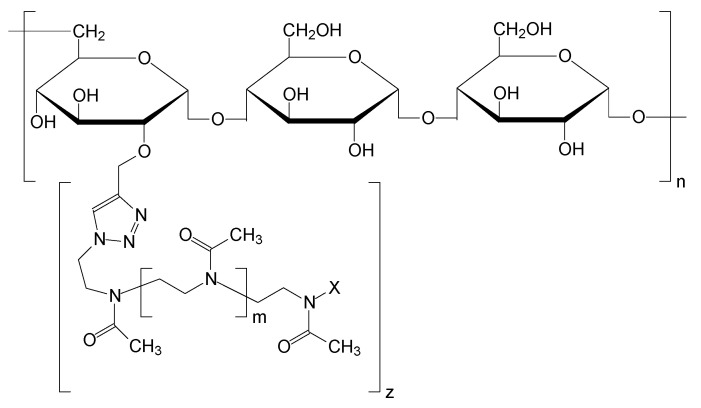
Chemical structure of pullulan-graft-poly(2-methyl-2-oxazoline). Here n, m—the degrees of polymerization of pullulan and polyoxazoline, respectively; X—initiator residue (−SO_2_-Ph-CH_3_ or −SO_2_-Ph-NO_2_); z—the degree of pullulan substitution.

In the present work, the modification of pullulan through poly(2-methyl-2-oxazoline) side chains of varying lengths and substitution numbers was successfully carried out using a click chemistry approach. The conditions of efficient and non-labor-intensive synthesis with a controlled degree of modification were drawn up. The resulting graft copolymers were characterized using the classical molecular hydrodynamics approach and nuclear magnetic resonance (NMR) spectroscopy. The chemical structure of the obtained copolymers is depicted in Figure 1. For simplicity, one of the possible points of grafting is shown, while in fact any (or all) of the hydroxyl groups of pullulan could be substituted.

## 2. Results and Discussion

### 2.1. Poly(2-methyl-2-oxazoline)

To synthesize a graft copolymer by means of the “grafting-to” method via azide–alkyne cycloaddition, azide-terminated poly(2-methyl-2-oxazoline)s (PMeOx) were obtained via living cationic polymerization of 2-methyloxazoline in sulfolane using 4-nitrophenylsulfochloride as an initiator. Varying the monomer/initiator ratio with other conditions equal, polyoxazolines with different molecular weights were obtained. To form azide groups, polymerization was terminated with a saturated (C = 40 g/L) sodium azide solution in DMSO. Polymers were dialyzed against water and lyophilized. In the ^1^H NMR spectrum of the product (Figure S1), in addition to the signals of protons of methylene and methyl groups of the main chain, signals of the end 4-nitrophenyl group and the pre-end units of the azide and nitrophenyl end of the chain are clearly visible. This indicates that the target polymer was obtained. The polymerization degree of the product was determined from the NMR data using the ratio of the integral intensity of the signals of protons of the end groups and protons of the main chain. The data of the calculated molecular weights are presented in Table 1 together with gel permeation chromatography (GPC) data.

Furthermore, the molar mass was also determined through the sedimentation/diffusion analysis using the Svedberg equation:

$$M_{sD}=\frac{s_{0}RT}{D_{0}(1-\bar{v}\rho_{0})}$$

where *M_sD_* is molecular mass; *D*_0_ and *s*_0_ are the diffusion and sedimentation coefficients, respectively, within the infinite dilution limit; $\bar{v}$ is the partial specific volume; $\rho_{0}$ is the solvent density; *T* is the temperature in K; *R* is the universal gas constant.

**Figure 2:**
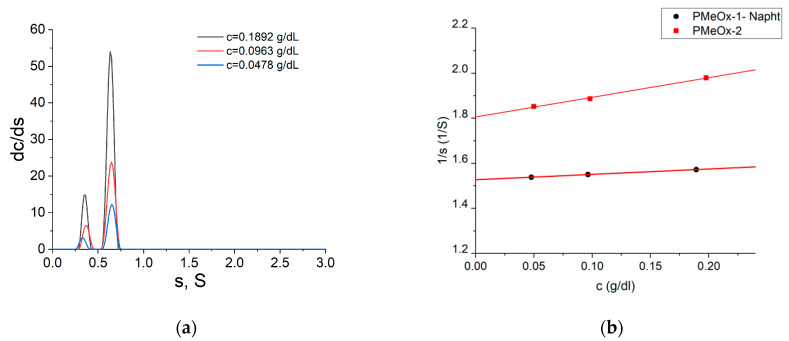
(**a**) Differential distributions of the sedimentation coefficients resolved with Sedfit for PMeOx-2 in water at 25 °C for three concentrations; concentration values are indicated in the figure. (**b**) Concentration dependencies of the inverse sedimentation coefficients for PMeOx-1 with naphthyl end groups (PMeOx-1-Napht) and PMeOx-2.

The combination of sedimentation and diffusion coefficients obtained independently results in the “absolute” molar mass value. The differential distributions of the sedimentation coefficients for PMeOx-2 are shown in Figure 2a, and corresponding concentration dependencies of the sedimentation coefficients for both studied samples are shown in Figure 2b.

It can be discerned that the distribution of sedimentation coefficients is bimodal. This could be a consequence of the presence of macromolecules with a lower polymerization degree. Considering the presence of a smaller component, the percentage of the main component can be estimated and is about 80%; thus, this mode was chosen to determine *s*_0_. Intrinsic viscosities ([*η*]) were determined using the Huggins and Kraemer extrapolation procedures (Figure S2). The diffusion coefficients were obtained using dynamic light scattering (DLS).

The self-consistency of determined hydrodynamic parameters and molar mass was established using the concept of the Tsvetkov–Klenin hydrodynamic invariant [50,51]:

$$A_{0}=\frac{\eta_{0}D_{0}{\left(M[\eta ]\right)}^{1/3}}{T}$$

where *M* is the molar mass, *η*_0_ is the solvent viscosity, and [*η*] is the intrinsic viscosity. The obtained values are shown in Table 2. The average value of *A*_0_ = (3.14 ± 0.22) × 10^−10^ g cm^2^/(s^2^K mol^1/3^) is typical for flexible-chain and semi-rigid polymers in good and theta solvents and is close to the one obtained earlier in a wide molar mass range series of poly(2-methyl-oxazoline)s [36,52]. Thus, the mutual agreement of the characteristics was proved, and the determination of molar masses using the Svedberg equation is possible. Molecular hydrodynamic characteristics of poly(2-methyl-oxazoline) samples obtained in water at 25 °C accompanied by molar mass *M_sD_* are listed in Table 2.

The previously determined partial specific volume of poly(2-methyl-2-oxazoline): *ῡ*_pMeOx (2022)_ = 0.804 cm^3^/g [52].

The accurate determination of the molecular mass of the poly(2-methyl-oxazoline)s is required for the more reliable determination of the degree of substitution of the resulting graft copolymers. At the same time, the results obtained through molecular hydrodynamic methods make it possible to distinguish between remaining and “grafted” poly(2-methyl-oxazoline)s in the mixture after the synthesis of graft copolymers and thus draw conclusions about the results of the grafting procedure.

**Figure 3:**
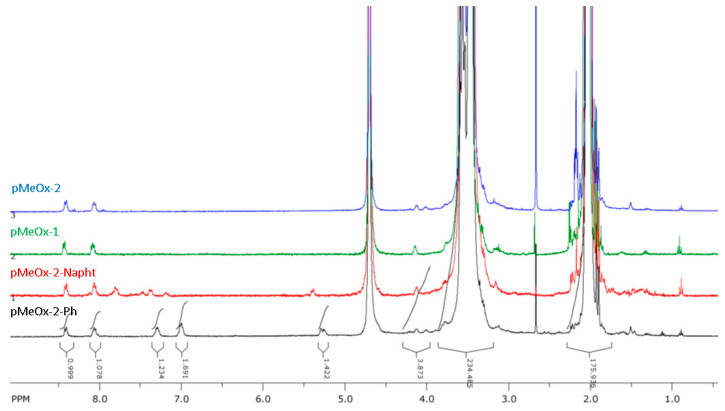
NMR spectra in D_2_O of polyoxazolines pMeOx-1, pMeOx-2 and their phenyloxy- and naphthyloxy-derivatives.

Model reactions of the azide-end-capped polyoxazoline with propargyloxybenzene and propargyloxynaphthalene (a mixture of isomers) were carried out to verify the presence and reactivity of the azide end group and additional calculations of M_n_ from NMR data. After the click reaction, the polymer was isolated by means of dialysis using a 2000 MWCO membrane. In the NMR spectra (Figure 3, Figure S3) of azide-end-capped polyoxazolines, the signals from the initiator groups (nitrophenyl) and terminal (phenyloxy-) groups corresponded to 1:1 mole fractions of the groups, and the degree of polymerization of the polymer calculated from NMR data coincided with the degree of polymerization of the starting polymer.

### 2.2. Pullulan

In this work, commercial pullulan (cosmetics/food grade) was used with a molecular weight of 120,000 g/mol and humidity of 7%. The NMR spectrum of the starting pullulan is given in Figure S4, and it is practically identical to the NMR spectrum of pullulan supplied by Sigma [53]. To synthesize propargyl pullulan, a modified method from [54] was used. For the reaction, mixed water/DMSO solvent was used with quite a high concentration of KOH for super-basic conditions. In such a solvent, native pullulan is not soluble but swells and forms a gel. Propargyl bromide as an 80% toluene solution was added in several portions to this mixture. Inhomogeneous conditions did not retard the reaction as indicated in [54]. The progress of the reaction was accompanied by strong gelation in the first 10–30 min followed by a gradual viscosity decrease in the next 2–3 h. After being stirred overnight, the reaction was stopped by the extraction of toluene and residual propargyl bromide with petroleum ether and quick washing out of KOH with water and ethanol. After dialysis against water and freeze-drying, propargyl pullulan was isolated as white wool. By varying the ratio of pullulan/KOH/propargyl bromide, two different propargyl pullulans (Pu-1, Pu-2) with a degree of substitution of 1/25 and 1/13, respectively, were obtained.

**Figure 4:**
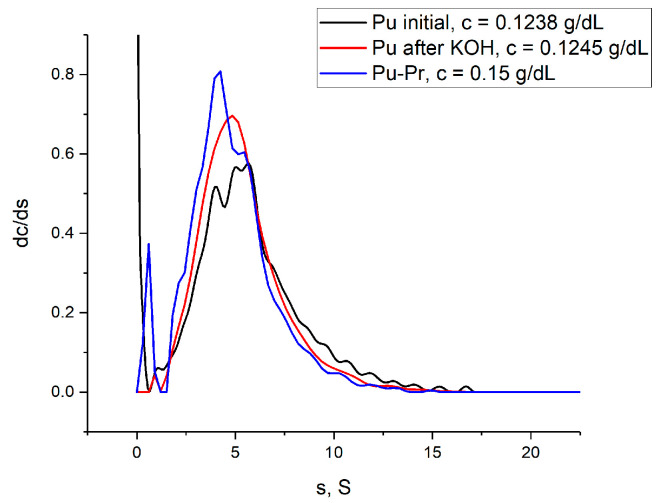
Differential distributions of the sedimentation coefficients resolved with the continuous distribution c(s) Sedfit model for the pullulan samples: initial Pu, Pu after KOH/DMSO treatment and propargyl–pullulan. The data were obtained in water at 25 °C; concentration values are indicated in the figure.

To evaluate the impact of reaction conditions on pullulan chains, a blank experiment without added propargyl bromide was performed. For the analysis, velocity sedimentation experiments were performed. The differential distributions of sedimentation coefficients for the initial pullulan, pullulan treated with KOH, and propargyl–pullulan are shown in Figure 4.

An observable broadening of the peak and a shift of its maximum to the region of lower sedimentation coefficients indicates slight degradation of pullulan polymer chains. However, we assume the difference appeared to be not crucial for our means.

The estimated parameters are listed in Table 3. The molar masses *M_sf_* were estimated using analytical ultracentrifugation (AUC) data according to the following equation:

$$M_{sf}=9\pi \sqrt{2}N_{A}{\left(\left[s\right]f/f_{0}\right)}^{3/2}\sqrt{\upsilon },$$

where $[s]=\frac{s_{0}\eta_{0}}{\left(1-\upsilon \rho_{0}\right)}$ is the intrinsic sedimentation coefficient; *N_A_*—Avogadro’s number.

The sedimentation coefficients, *s*, and frictional ratios, *f*/*f*_0,_ were obtained using a single measurement.

The accurate determination of the sedimentation coefficients s_0_ and thus molar masses requires obtaining a concentration dependence of sedimentation coefficients and frictional ratios or diffusion coefficients. Nonetheless, the estimation of the sedimentation coefficient, frictional ratio, and thus molar mass can be made using a single measurement, assuming that the concentration dependence in the region of small concentrations is not strong. For example, such an estimation of the molar mass of the initial pullulan using data for the solution at concentration c = 0.1238 g/dL results in *M_sf_* = 119,000 g/mol vs. 120,000 g/mol obtained through 3 concentrations and 117,000 g/mol obtained from SEC. The difference here is within the experimental error. 

**Figure 5:**
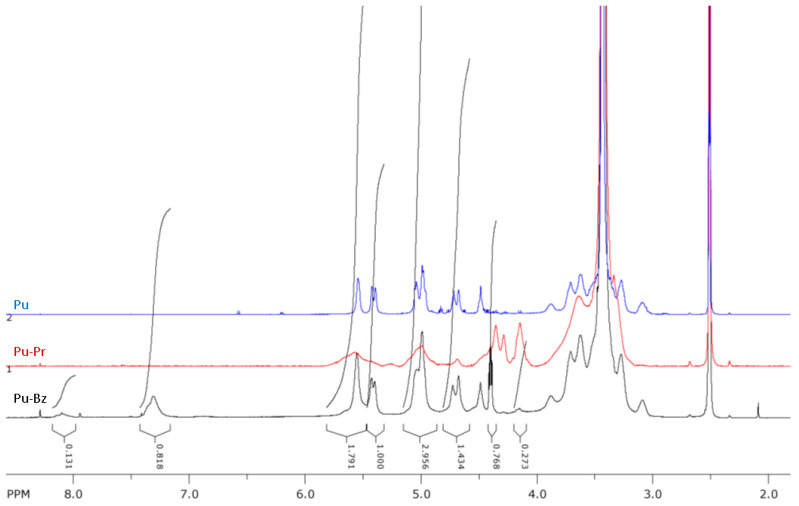
NMR spectra in DMSO-d6 of the starting pullulan (Pu), proparyl pullulan (Pu-Pr), and its benzyl derivative (Pu-Bz).

The changes in NMR spectra after the propargylation of pullulan appeared to be not clear to identify the propargyl group and the degree of substitution. Thereby, we performed a model click reaction of the obtained propargyl pullulan with benzyl azide. The NMR spectrum of the reaction product after purification clearly indicates the presence of benzyl and triazole groups attached to the pullulan chain and makes it possible to calculate the degree of substitution. In the spectrum (Figure 5, Figure S5), the degree of substitution was 1/18—every 18th ring of the pullulan chain is substituted (or about 1/6 of pullulan repeating units). Propargyl–pullulan with such a degree of substitution is still soluble in water, but the increase in the contents of propargyl or benzyl groups to 1/13 and higher leads to a water-insoluble polymer, which is soluble in DMSO.

### 2.3. Pullulan-Graft-Poly(2-methyl-2-oxazoline)

**Figure 6:**
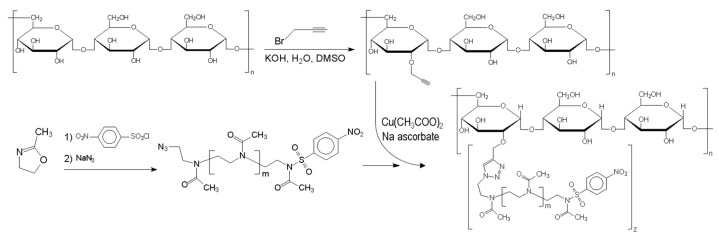
Scheme of the pullulan-graft-poly(2-methyl-2-oxazoline) synthesis.

Click reaction of propargyl–pullulan with polyoxazoline-azide was performed in water or DMSO with sodium ascorbate and copper (ii) acetate as a catalyst for 1 to 3 days at room temperature (RT). At least 10–20-fold by mass and a higher ratio of polyoxazoline to pullulan (which corresponds to the ratio of one polyoxazoline chain per 1 to 10 pullulan rings) was used to achieve various degrees of substitution. Unreacted polyoxazoline was removed by prolonged dialysis in a 12,000 MWCO membrane and reprecipitation. The scheme illustrating and explaining the sequence of the synthesis is presented in Figure 6.

The obtained samples are marked as Pu-x-PMeOx-y, where x is the number of propargyl pullulan and y is the number of the PMeOx sample, Pu-x-Bz is pullulan with benzyl side groups.

**Figure 7:**
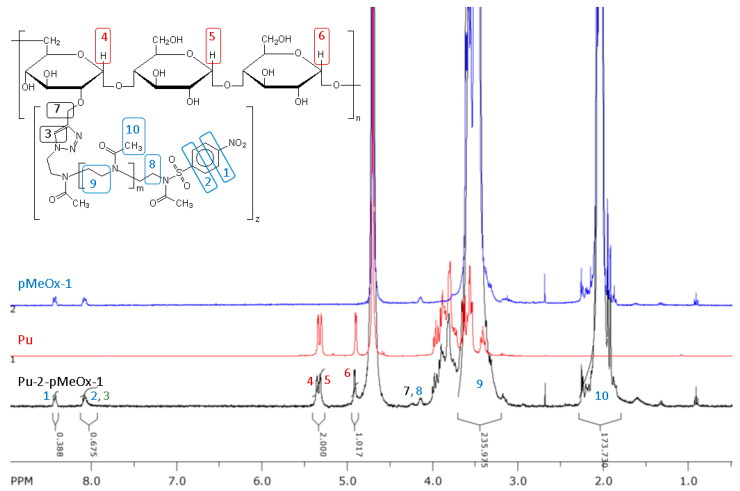
^1^H NMR spectra in D_2_O of pullulan (Pu), polyoxazoline (pMeOx-1) and pullulan-graft-polyoxazoline (Pu-2-pMeOx-1).

In the NMR spectra of pullulan-graft-polyoxazoline recorded in D_2_O (Figure 7), the signals of pullulan anomeric protons (three types, 1:1:1 to each other) are quite clear and suitable for calculations, as well as signals of the polyoxazoline chain and side groups (methylene and methyl groups, 4:3 to each other). Also, signals from polyoxazoline end groups are clearly visible (both ends—nitrophenyl from the initiator and triazole from azide–alkyne addition, 4:1 to each other). The observed integral intensities of the corresponding signals conform to the expected chemical structure and composition of the graft copolymer.

The degrees of substitution were calculated from NMR spectra using the ratio of the integral intensities of signals of pullulan anomeric protons and (a) polyoxazoline end groups (nitrophenyl) or (b) a polyoxazoline chain of known length. Basically, results from the a and b methods were concordant for fresh polyoxazoline samples but some hydrolysis of nitrophenyl end groups of polyoxazoline may influence the results, so the calculations based on the predetermined polyoxazoline molecular weight are more reliable.

So, we estimated the degree of substitution by utilizing NMR data and molar masses of grafted poly(2-methyl-2-oxazoline) molecules obtained through molecular hydrodynamic methods. The polyoxazoline chain/glucose ring ratio in the purified samples ranged from 1/50 to 1/13 (chains per ring), as indicated in Table 4.

Graft copolymers possess a level of solubility that is uncommon for intact pullulan: besides water, DMF, and DMSO, pullulan-graft-PMeOx appeared to be soluble in alcohols, and at a high degree of substitution—in chloroform and methylene chloride—and remain insoluble in diethyl ether, acetone, THF, and ethyl acetate. The Pu-2-Bz sample appeared to be insoluble in water; thus, DMF was chosen as a solvent for the analysis of molar masses both for Pu-1-Ph and its derivatives and Pu-2-Ph and its derivatives.

A detailed analysis of the graft copolymers revealed a considerable decrease in the molecular weight of the pullulan chain compared to the starting polymer, as well as compared to propargyl pullulan and KOH-treated pullulan. At the same time, all samples of the copolymers obtained contained a certain amount (up to 50%) of a component having molecular weight 20–50% higher than grafted PMeOx (about 12,000 g/mol while the PMeOx-1 molar mass in DMF was determined to be 8000 g/mol, PMeOx-2—around 4700 g/mol). This component appeared to be identical to the graft copolymer in NMR and not separated during dialysis and reprecipitation. This may indicate that during the Cu-catalyzed click reaction, some degradation of the pullulan chain may occur, and a certain number of the pullulan chain fragments bound to the polyoxazoline chain may be formed with the composition of 10 to 20 saccharide units per polyoxazoline chain. The chemistry of this process may include oxidative degradation of the pullulan chain as described in [55] and should be the subject of a separate investigation. In previous works on block copolymers [40,41], no pullulan degradation under conditions of Cu(ii)/ascorbate click reaction was reported by means of SEC, which might be due to a chromatographic effect, for example, interactions with the column, but also might have a connection to the fact that already partly depolymerized pullulan was used.

The molar masses *M_sf_* of the obtained pullulan derivatives, estimated using the approach described above, are given in Table 4 with the accompanying parameters. The value of the partial specific volume increases with an increase in the degree of substitution, reaching the value of the partial specific volume of poly(2-methyl-2-oxazoline)s. The concept of the hydrodynamic invariant A_0_ was applied to check the self-consistency of the experimental data. Keeping in mind that the listed molar masses *M_sf_* are an estimation, one should note that the A_0_ value belongs to the region characteristic for the polymer chains in good solvents and thus sedimentation and viscometry data are in satisfactory agreement with each other. The differential distributions of the sedimentation coefficients *s* for the studied samples are shown in Figure S6. The molar masses of Pu-PMeOx samples compared to Pu-Bz samples increase in all cases, which indirectly indicates the grafting of the side chains. At the same time, the wide molar mass distribution and the aforementioned degradation processes do not make it possible to determine the exact degree of substitution using AUC data. On the other hand, the molar mass increases with an increase in the degree of substitution (comparing Pu-1-PMeOx-2 [1/50] and Pu-1-PMeOx-2 [1/3.4]).

**Figure 8:**
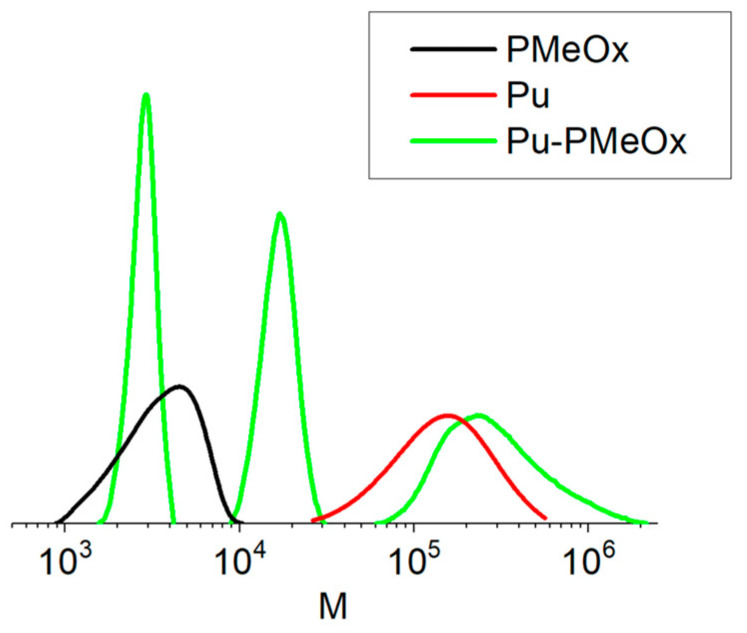
SEC results for poly(2-methyl-2-oxazoline), starting pullulan, and the product of click reaction.

Size exclusion chromatography (Figure 8) also clearly indicates that both processes—grafting and pullulan chain degradation take place under conditions of Cu(ii)-ascorbate click reaction. In the reaction product, a high-molecular-weight substance (M_n_ = 235,000 g/mol) is detected which should be the desired graft copolymer. Two additional signals are present in the chromatogram, one can be attributed to unreacted PMeOx, but the middle one (M_n_ = 16,300 g/mol) can correspond to the product of both pullulan degradation and PMeOx grafting to it and can contain 2 to 3 chains of PMeOx and 50 to 70 saccharide units. It should additionally be noted that all products of the grafting reaction appeared as homogeneous in dissolution, precipitation, and dialysis processes and mostly comply with the concept of “polymer” rather than “mixture of substances”.

## 3. Materials and Methods

### 3.1. Materials

Pullulan (cosmetology grade, Terra-aromatica, Moscow, Russia), propargyl bromide (80% in toluene), and sodium azide (Acros Organics, Antwerp, Belgium), 4-nitrobenzeneulfonylchloride (Thermo Scientific, Waltham, MA, USA), sodium ascorbate, and copper (ii) acetate were used as received. Dimethyl sulfoxide (DMSO) and sulfolane were distilled under reduced pressure, dimethylformamide (DMF) was distilled under reduced pressure over calcium hydride. 2-Methyloxazoline was kept for 24 h with sodium metal and then distilled over fresh sodium under argon.

Pullulan purchased was characterized by NMR (Figure S4), viscometry, densitometry, and AUC methods in water at 25 °C, and the determined parameters were the following: partial specific volume *ῡ* = 0.65 cm^3^/g, intrinsic viscosity [*η*] = (0.68 ± 0.02) dL/g, and estimated average molar mass based on velocity sedimentation data *M_sf_* = 120,000 g/mol. The differential distributions of the sedimentation coefficients for three concentrations are shown in Figure S7a and the concentration dependence of inverse sedimentation coefficients is shown in in Figure S7b.

The solutions for intrinsic viscosity, density, velocity sedimentation, and DLS measurements were prepared using ultrapure (Type 1) water obtained using the Direct-Q^®^ 8 UV Water Purification System (Merck KGaA, Darmstadt, Germany) or DMF. Solvent viscosity and density at 25 °C, respectively: (i) water—*η*_0_ = 0.89 cP, *ρ*_0_ = 0.9971 g/cm^3^; (ii) DMF—*η*_0_ = 0.805 cP, *ρ_0_* = 0.9448 g/cm^3^.

### 3.2. Methods

#### 3.2.1. Nuclear Magnetic Resonance (NMR) Spectroscopy

^1^H-NMR spectra were recorded on a Bruker 400 MHz Avance spectrometer in DMSO-d6, CDCl_3_, and D_2_O at T = 25 °C.

#### 3.2.2. Densitometry

Partial specific volume was determined through density measurements. Solution density measurements were carried out at 25 °C using a density meter DMA 5000 M (Anton Paar GmbH, Graz, Austria) according to the procedure described in [56].

#### 3.2.3. Viscometry

Intrinsic viscosities were determined at 25 °C by standard dilution procedures [57] via Huggins [58] and Kraemer [59] plots. Viscosity measurements were performed at 25 °C using a rolling ball microviscometer Lovis 2000 M (Anton Paar GmbH, Graz, Austria). The setup included a capillary with an inner diameter of 1.59 mm and equipped with a gold-coated steel ball (1.50 mm in diameter), and the chosen capillary inclination angle was 45°.

#### 3.2.4. Size Exclusion Chromatography/Gel Permeation Chromatography (SEC/GPC)

SEC measurements were performed on Shimadzu LC-20AD, equipped with a TSKgel G5000HHR column (Tosoh Bioscience, San Francisco, CA, USA) in 0.1 M LiBr/DMF solution at 60 °C and 0.5 mL/min rate and with a set of columns PROTEEMA 50 × 8 mm, 5 μm (PSS); PROTEEMA 300 × 8 mm, 300 Å (PSS); PROTEEMA 300 × 8 mm, 1000 Å (PSS) in water (with 0.05% sodium azide) at 30 °C and 1.0 mL/min rate. Polymer solutions (3 g/L) were filtered through a PTFE membrane, 0.22 μm. Column calibration was performed using polystyrene (M = 500–250,000 g/mol) or pullulan (M = 6300–708,000 g/mol) standards.

#### 3.2.5. Analytical Ultracentrifugation (AUC)

Velocity sedimentation experiments were performed at 25 °C with a ProteomeLab XLI Protein Characterization System analytical ultracentrifuge (Beckman Coulter, Brea, CA, USA) using double-sector cells with aluminum centerpieces with an optical path length of 12 mm, and a four-hole analytical rotor (An-60Ti) was used. The rotor speeds were 50,000–55,000 rpm depending on the sample. The sample and the reference sectors were loaded with 0.42 mL of the studied solution and 0.44 mL of a solvent, respectively. Sedimentation profiles were obtained using the interference optical system equipped with a red laser (λ = 655 nm) as a light source. The centrifuge chamber with a loaded rotor and interferometer was vacuumed and thermostated for at least 1.5 h before the run.

The velocity sedimentation data analysis was processed using the Sedfit software (Version 16.1c) [60]. Sedimentation coefficients here are obtained through the numerical solution of the Lamm equation [61]. The continuous c(s) distribution model implemented within Sedfit coupled with a Tikhonov–Phillips regularization procedure makes it possible to obtain the differential distribution on the sedimentation coefficients s and the frictional ratio (*f*/*f*_0_), where *f*_0_ is a translation friction coefficient of the equivalent sphere (a spherical particle having the same size and the density as the studied one). The averaged diffusion coefficient *D*_0_ can then be reestablished using the velocity sedimentation data as:

$$D_{0}=\frac{k_{B}T{\left(1-\upsilon \rho_{0}\right)}^{1/2}}{\eta_{0}^{3/2}9\pi \sqrt{2}{\left(f/f_{0}\right)}^{3/2}{\left(s_{0}\upsilon \right)}^{1/2}}$$

where *k_B_* is the Boltzmann constant; *T* is the temperature in K; *ῡ*—partial specific volume; $\rho_{0}$—the density of the solvent; *η*_0_—the dynamic viscosity of the solvent; *s*_0_—the sedimentation coefficient; *f/f*_0_—the frictional ratio.

The estimated sedimentation and diffusion coefficients together with the determined partial specific volumes can be used for the calculation of the molar masses via the Svedberg equation. Thus, the molar mass calculation can be represented as

$$M_{sf}=\frac{RTs_{0}}{\left(1-\upsilon \rho_{0}\right)D_{0}}=9\pi \sqrt{2}N_{A}{\left(\left[s\right]f/f_{0}\right)}^{3/2}\sqrt{\upsilon },$$

where $[s]=\frac{s_{0}\eta_{0}}{\left(1-\upsilon \rho_{0}\right)}$ is the intrinsic sedimentation coefficient; *N_A_*—Avogadro’s number.

#### 3.2.6. Dynamic Light Scattering (DLS)

The diffusion coefficients were determined using the DLS data.

DLS experiments were performed at 25 °C using a PhotoCor Complex spectrometer (Photocor Instruments Inc., Moscow, Russia) with a standard goniometer (10°–150°), digital correlator (288 channels, 10 ns), and a thermostat with temperature stabilization of 0.05 °C. A single-mode linear polarized laser (λ = 405 nm) was used as an excitation source; the experiments were carried out at scattering angles ranging from 30° to 130°. Autocorrelation functions of scattered light intensity were processed using the DynaLS software (Version 2.7.1, Photocor Instruments Inc., Moscow, Russia) using the inverse Laplace transform regularization procedure [62,63,64].

### 3.3. Synthesis of Precursor Polymers and Graft Copolymers

#### 3.3.1. Azide-Terminated Poly(2-methyl-2-oxazoline)

Measures of 2 mL of methyloxazoline, 2 mL of sulfolane, and 40 mg of 4-nitrobenzeneulfonylchloride were placed in a 10 mL vial with septa. The vial was evacuated to 10 mmHg and filled with argon (three cycles). The evacuated vial was placed in a bath with T = 95 °C with magnetic stirring. After 20 h, the reaction mixture was cooled to RT, and 1.5 mL of the saturated solution of sodium azide in DMSO (40 g/L) was added and left overnight. The polymer was isolated from the reaction mixture by dialysis against water in a 2000 MWCO membrane and freeze-dried. Yield 1.75 g. M_n_ (NMR, Figure S1) 11,700 g/mol; *M_sD_* = 10,000 g/mol, X_sD_ = 118.

Then, 3.5 mL of methyloxazoline, 3.5 mL of sulfolane, and 185 mg of 4-nitrobenzeneulfonylchloride were placed in a 10 mL vial with septa. The vial was evacuated to 10 mmHg and filled with argon (three cycles). The evacuated vial was placed in a bath with T = 95 °C with magnetic stirring. After 20 h, the reaction mixture was cooled to RT, and 1.5 mL of the saturated solution of sodium azide in DMSO (40 g/L) was added and left overnight. The polymer was isolated from the reaction mixture by dialysis against water in a 2000 MWCO membrane and freeze-dried. Yield 3.3 g. M_n_ (NMR) 6800 g/mol; *M_sD_* = 4600 g/mol, X_sD_ = 54.

#### 3.3.2. Phenyloxy Poly(2-methyl-2-oxazoline), Naphthyloxy Poly(2-methyl-2-oxazoline) (PMeOx-Ph, PMeOx-Napht)

To a solution of poly(2-methyl-2-oxazoline)-azide in DMSO propargyloxybenzene (or propargyloxynaphthalene), sodium ascorbate and copper (ii) acetate (2–3 mg both) were added, and the mixture was allowed to stir at RT for several days. The reaction mixture was diluted with water, washed with hexane, dialyzed against water in a 2000 MWCO membrane, and freeze-dried (NMR, Figure S3).

#### 3.3.3. Propargyl Pullulan (Pu-Pr)

To a solution of 4.43 g potassium hydroxide in 6.1 mL of water and 5.5 mL DMSO, 1.6 g of pullulan was added under stirring with a big magnetic stirrer. After 10 min, 4 mL of propargyl bromide (80% in toluene) was added to this inhomogeneous mixture in 1 mL portions in two minutes. The reaction mixture became very viscous in about 20 min, and then viscosity was gradually decreased in 2–3 h. Stirring was continued overnight at RT; after that, the reaction mixture was washed with petroleum ether (thoroughly, 40 mL twice), water (quickly, 30 mL twice), and ethanol (thoroughly, 50 mL twice). After washing, it was dissolved in water, dialyzed against water (membrane 12,000 MWCO), and freeze-dried. By varying the pullulan/KOH/propargyl bromide ratio, two different propargyl pullulans (Pu-1, Pu-2) were obtained with substitution degrees of 1/25, 1/13.

Propargyl pullulan is soluble in DMSO, DMF, and water if the degree of substitution is not high.

#### 3.3.4. Benzyl Pullulan

To a solution of propargyl pullulan in DMSO, benzyl azide, sodium ascorbate, and copper (ii) acetate were added, and the mixture was allowed to stir at RT for several days. Then, the product was isolated by precipitation in plenty of ethanol, filtered, washed with ethanol, additionally dialyzed sequentially against DMF and water, and freeze-dried. The degree of substitution was calculated from NMR, Figure S5.

#### 3.3.5. Pullulan-Graft-Poly(2-methyl-2-oxazoline)

To a solution of propargyl pullulan (20–50 mg) in water or DMSO, azide-terminated poly(2-methyl-2-oxazoline) (300–700 mg), sodium ascorbate and copper (ii) acetate were added, and the mixture was allowed to stir at RT for several days. Then, the product was dialyzed against water (12,000 MWCO membrane) and freeze-dried. The product after dialysis still contained some amount of unreacted polyoxazoline, which was removed via reprecipitation from methanol to ethylacetate.

## 4. Conclusions

New pullulan derivatives, namely pullulan-graft-poly(2-methyl-2-oxazoline) with different degrees of substitution, were obtained via the click chemistry approach. The fact of grafting was confirmed independently via NMR and molecular hydrodynamic methods. The degree of substitution was determined using a combination of the accurately defined molecular weight of side chains and NMR data. In all cases, a decrease in the pullulan molecular weight was detected and all samples contained up to 50% short pullulan chain fragments bound to polyoxazoline macromolecules. These by-products seem to be formed in the stage of Cu(ii)-ascorbate-mediated click reaction.

Thus, the modification of pullulan by poly(2-methyl-2-oxazoline) side chains of varying lengths and substitution numbers was carried out using the click chemistry approach. This demonstrated a method of modifying polysaccharides with poly(2-oxazoline) side chains, highlighting all the pros and cons of this approach.

After optimizing Cu-AAc conditions, in order to make it possible to receive poly(2-oxazolines) grafted to high-molecular-weight polysaccharides (pullulan, gellan) without the destruction of the latter, new possibilities arise for creating novel polymer systems with the potential of application in targeted drug delivery, for example, in ophthalmology.

## Figures and Tables

**Table 1:** The molecular weights of the obtained poly(2-methyl-2-oxazoline)s determined through NMR, GPC, and sedimentation/diffusion analysis—*M_sD_*.

Sample	*M_n_* (NMR), g/mol	*M_n_* (GPC), g/mol	*M_w_* (GPC), g/mol	*M_sD_*, g/mol
PMeOx-1	11,700	7800	11,000	10,100
PMeOx-2	6800	6200	7800	4600

**Table 2:** Molecular hydrodynamic characteristics (partial specific volume *ῡ*, sedimentation coefficient *s*_0_, frictional ratio *f*/*f*_0_, intrinsic viscosity [*η*], diffusion coefficients *D*_0_), the resulting molar mass *M_sD_*, and the magnitude of the hydrodynamic invariant A_0_ of PMeOx samples in water at 25 °C.

Sample	*ῡ*, cm^3^/g	*s*_0_, S	*f*/*f*_0_	[*η*], dL/g	*D*_0_ × 10^7^, cm^2^/s	*M_sD_*, g/mol	*A*_0_ × 10^10^, g cm^2^/(s^2^K mol^1/3^)
PMeOx-1-Napht	0.805	0.70	1.66	0.14	8.70	10,100	2.92
PMeOx-2	0.797	0.55	1.40	0.10	14.4	4600	3.36

**Table 3:** Partial specific volume *ῡ*, sedimentation coefficient *s*, frictional ratio *f*/*f*_0_, the estimated molar mass *M_sf_* of the initial Pu, Pu after KOH/DMSO treatment, and propargyl–pullulan.

Sample	*ῡ*, cm^3^/g	*s*, S	*f*/*f*_0_	*M_sf_*, g/mol
initial Pu	0.65	6.3	2.1	119,000
Pu after KOH	0.62	5.1	3.02	129,000
Pu-Pr	0.62	5.01	2.68	105,000

**Table 4:** Partial specific volumes *ῡ*, sedimentation coefficients *s*, frictional ratios *f*/*f*_0_, estimated molar mass *M_sf_*, intrinsic viscosities [*η*], and the hydrodynamic invariant of the obtained graft copolymers in DMF at 25 °C.

Sample	PU/PMeOx *	*ῡ*, cm^3^/g	*s*, S	*f*/*f*_0_	*M_sf,_* g/mol	[*η*], dL/g	*A*_0_ × 10^10^, g cm^2^/(s^2^K mol^1/3^)
Pu-1-Bz	-	0.67	5.3	1.9	65,000	0.33	4.1
Pu-1-PMeOx-2	1/50	0.72	5.7	2.1	110,000	0.42	4.0
Pu-1-PMeOx-2	1/3.4	0.804	6.5	1.8	160,000	0.30	3.9
Pu-1-PMeOx-1	1/10	0.79	5.3	1.8	116,000	0.27	3.9
Pu-2-Bz	-	0.65	3.5	2.1	37,000	0.35	3.9
Pu-2-PMeOx-1	1/5	0.804	3.3	1.8	61,000	0.165	3.3

* polyoxazoline chain/glucose ring ratio in the purified samples as determined from NMR combined data with M*_s_*_D_ of PMeOxs.

## Data Availability

The data on the synthesis and characterization of all compounds are stored at St. Petersburg State University.

## Supplementary Materials

The following supporting information can be downloaded at: https://www.mdpi.com/article/10.3390/molecules29010026/s1, Figure S1: ^1^H NMR spectrum of polyoxazoline-azide; Figure S2: The dependences of reduced viscosity η_sp/c_ and lnη_r_/c on concentration for poly(2-methyl-2-oxazoline)s in water at T = 25 °C; Figure S3: ^1^H NMR spectrum of phenyloxy-polyoxazoline; Figure S4: ^1^H NMR spectrum of the starting pullulan in D_2_O; Figure S5: ^1^H NMR spectrum of benzyl-pullulan; Figure S6: The differential dc/ds distributions vs. sedimentation coefficients s for the obtained graft copolymers; Figure S7: (a) The differential dc/ds distributions vs. sedimentation coefficients s for three concentrations of pullulan in water; (b) the concentration dependence of inverse sedimentation coefficients.
