# Supplementary material for: Pullulan-Graft-Polyoxazoline: Approaches from Chemistry and Physics
Source: Molecules. 2023 Dec 19;29(1):26. doi: 10.3390/molecules29010026 (PMC10780122; doi:10.3390/molecules29010026)
Supplement: Supplementary file 1 [file molecules-29-00026-s001.zip › molecules-2750695-supplementary.pdf]

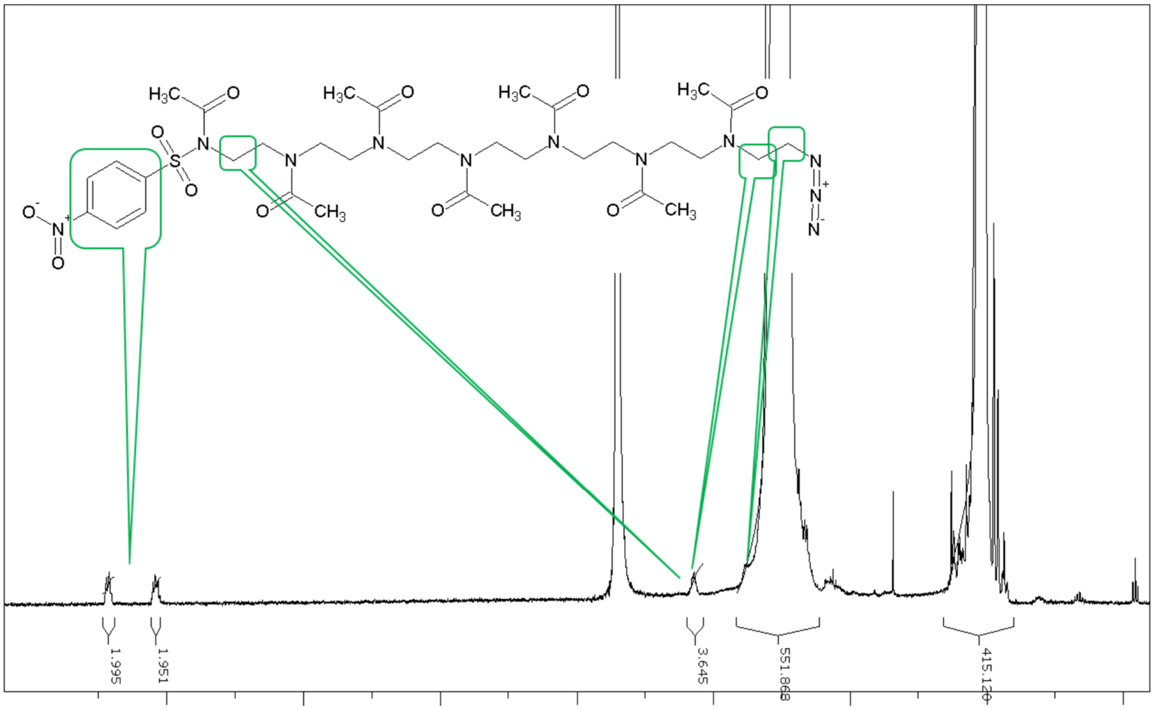

Figure S1. ^{1}H NMR spectrum of polyoxazoline-azide.

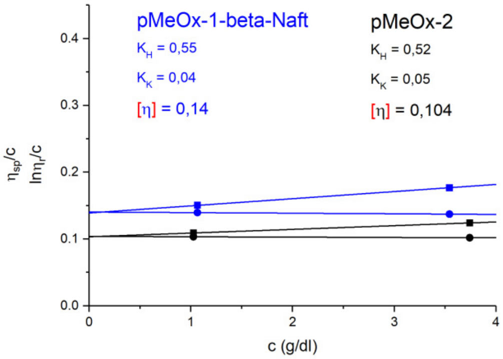

Figure S2. The dependences of reduced viscosity η_{sp}/c and $\ln\eta_r/c$ on concentration for poly(2-methyl-2-oxazoline)s in water at T = 25 °C.

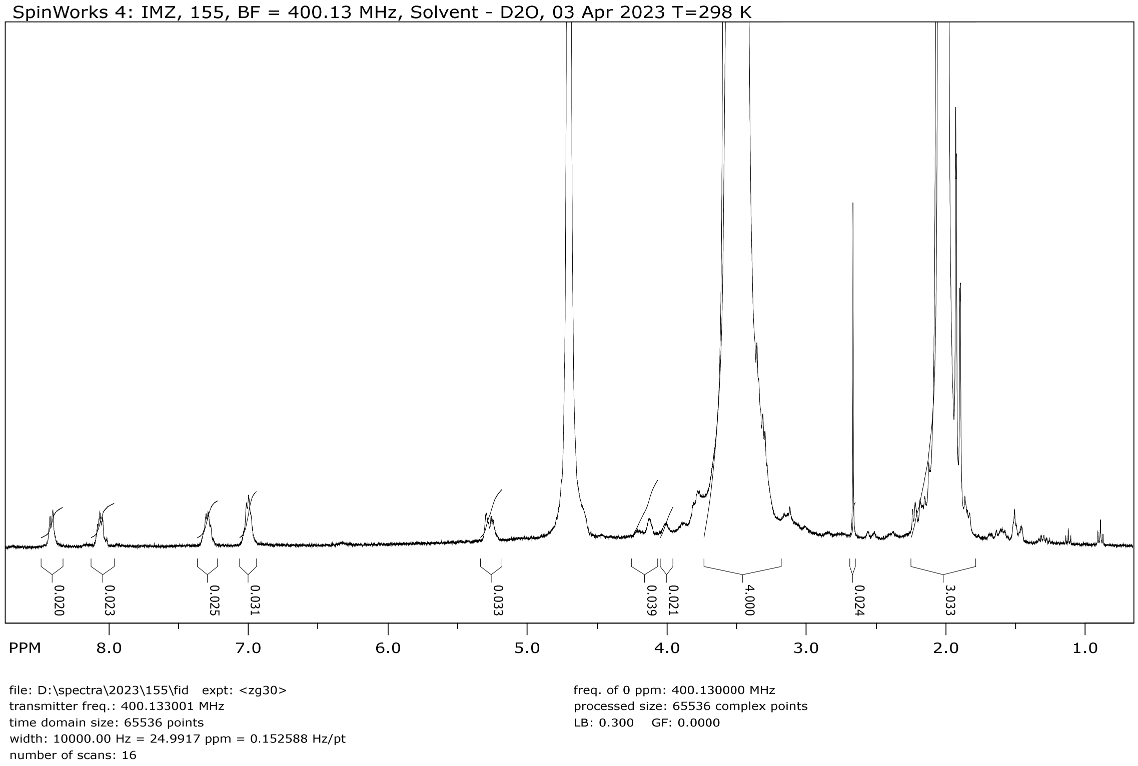

Figure S3. ^{1}H NMR spectrum of phenyloxy-polyoxazoline.

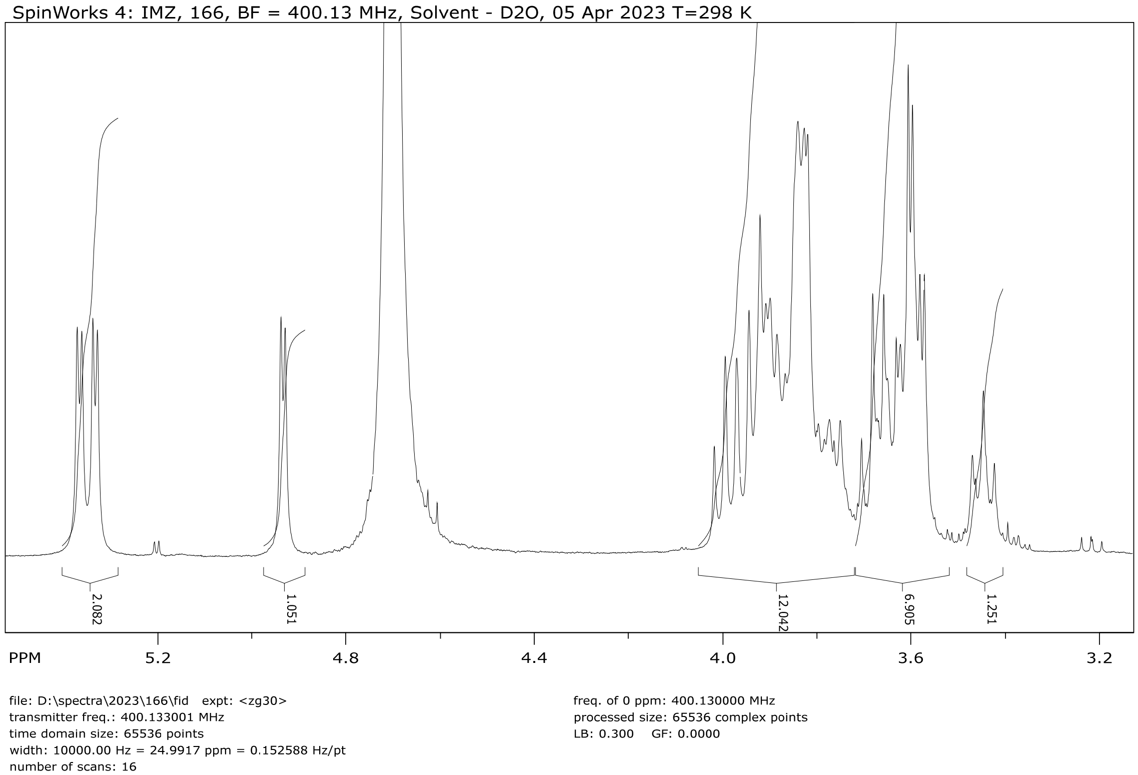

Figure S4. ^{1}H NMR spectrum of starting pullulan in D_2O.

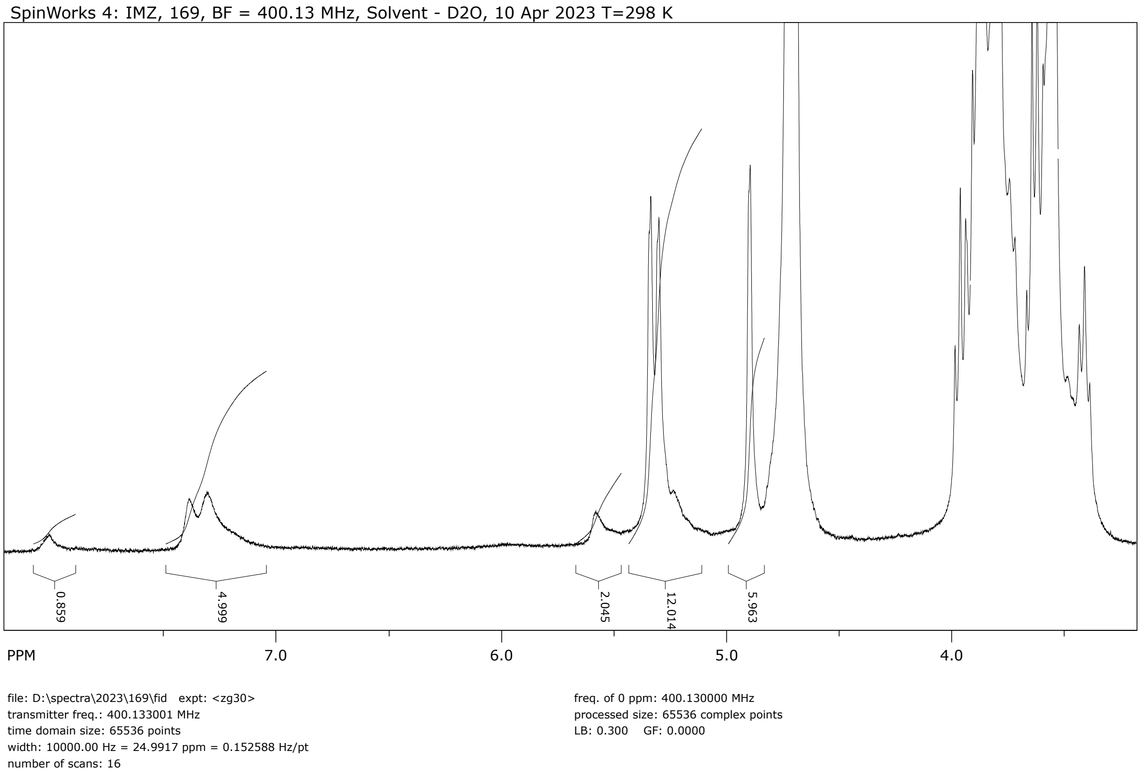

Figure S5. ^{1}H NMR spectrum of benzyl-pullulan.

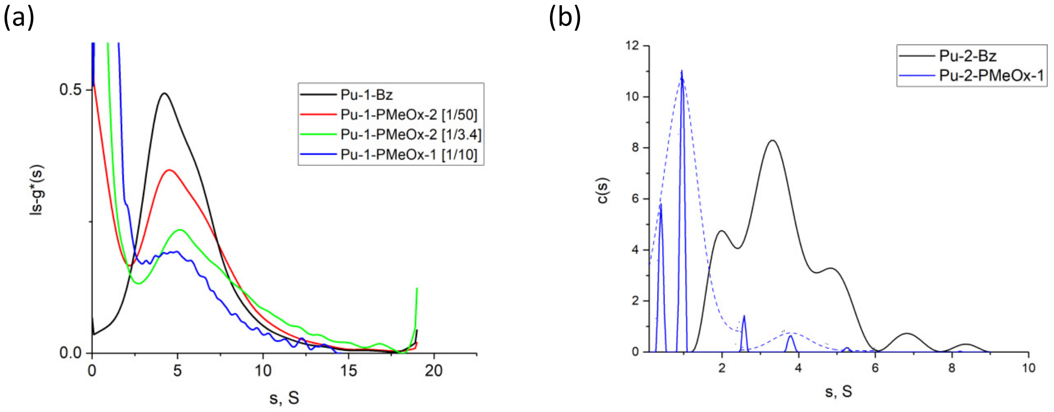

Figure S6. (a) The differential dc/ds distributions obtained using ls-g*(s) model in Sedfit *vs*. sedimentation coefficients s for solutions of samples: Pu-1-Bz (c = 0,1384 g/dl), Pu-1-PMeOx-2 [1/50] (c = 0,1584 g/dl), Pu-1-PMeOx-2 [1/3.4] (c = 0,15 g/dl), Pu-1-PMeOx-1 [1/10] (c = 0,15 g/dl) in DMF at 25°C. (b) The differential dc/ds distributions obtained using c(s) model in Sedfit vs. sedimentation coefficients s for solutions of samples: Pu-2-Bz (c = 0,1550 g/dl), Pu-2-PMeOx-1 (c = 0,1420 g/dl) in DMF at 25°C. Dashed lines represent smoothed envelope of peaks integrated together.

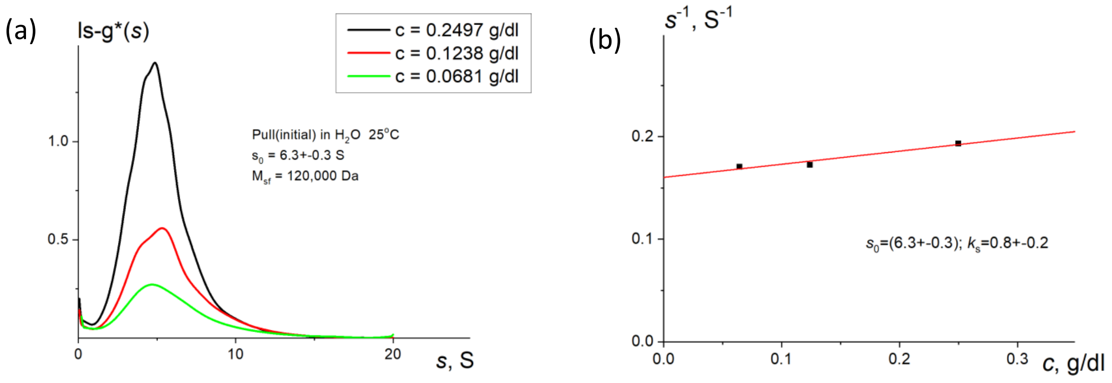

Figure S7. (a) The differential dc/ds distributions obtained using ls-g*(s) model in Sedfit vs. sedimentation coefficients s for three concentrations of pullulan in water (values given in the plot legend); (b) the concentration dependence of inverse sedimentation coefficients.
